# Use of Inotropic Agents in Treatment of Systolic Heart Failure

**DOI:** 10.3390/ijms161226147

**Published:** 2015-12-04

**Authors:** Sohaib Tariq, Wilbert S. Aronow

**Affiliations:** Cardiology Division, Department of Medicine, Westchester Medical Center, New York Medical College, Valhalla, New York, NY 10595, USA; sohaib.tariq@wmchealth.org

**Keywords:** inotropes, digoxin, dopamine, dobutamine, norepinephrine, milrinone, levosimendan, omecamtiv mecarbil

## Abstract

The most common use of inotropes is among hospitalized patients with acute decompensated heart failure, with reduced left ventricular ejection fraction and with signs of end-organ dysfunction in the setting of a low cardiac output. Inotropes can be used in patients with severe systolic heart failure awaiting heart transplant to maintain hemodynamic stability or as a bridge to decision. In cases where patients are unable to be weaned off inotropes, these agents can be used until a definite or escalated supportive therapy is planned, which can include coronary revascularization or mechanical circulatory support (intra-aortic balloon pump, extracorporeal membrane oxygenation, impella, left ventricular assist device, *etc.*). Use of inotropic drugs is associated with risks and adverse events. This review will discuss the use of the inotropes digoxin, dopamine, dobutamine, norepinephrine, milrinone, levosimendan, and omecamtiv mecarbil. Long-term inotropic therapy should be offered in selected patients. A detailed conversation with the patient and family shall be held, including a discussion on the risks and benefits of use of inotropes. Chronic heart failure patients awaiting heart transplants are candidates for intravenous inotropic support until the donor heart becomes available. This helps to maintain hemodynamic stability and keep the fluid status and pulmonary pressures optimized prior to the surgery. On the other hand, in patients with severe heart failure who are not candidates for advanced heart failure therapies, such as transplant and mechanical circulatory support, inotropic agents can be used for palliative therapy. Inotropes can help reduce frequency of hospitalizations and improve symptoms in these patients.

## 1. Introduction

Inotropic agents have been in use for many years for the treatment of patients with acute decompensated systolic heart failure, also known as heart failure with reduced left ventricular ejection fraction (HFrEF). These drugs improve the contractility of the myocardium by definition, but can also affect the heart rate and peripheral vascular resistance. The most common use of inotropes is among hospitalized patients with acute decompensated HFrEF with signs of end-organ dysfunction in the setting of a low cardiac output. Due to advancements in advanced heart failure therapies, including transplant and mechanical circulatory support, their use is becoming more common for other indications. Inotropes can be used in patients with severe systolic heart failure awaiting heart transplant to maintain hemodynamic stability, or as a bridge to decision. In cases where patients are unable to be weaned off inotropes, these agents can be used until a definite or escalated supportive therapy is planned, which can include coronary revascularization or mechanical circulatory support (intra-aortic balloon pump, extracorporeal membrane oxygenation, impella, left ventricular assist device, *etc.*).

Use of inotropic drugs is associated with risks and adverse events. Therefore, patient selection for short- and long-term use of these agents is important. In this review, we will discuss different inotropic agents that have been in use for decades, and some new drugs, with focus on their mechanism of action, indications, and evidence supporting their use Table 1.

**Table 1:** Inotropic drugs.

Drug	Mechanism	Increase in Intracellular Calcium Concentration	Effect on Mortality
Digoxin	Na-K pump inhibitor	Yes	Neutral; increased mortality of discontinued after long-term use [1]
Dobutamine	Pure adrenergic; β1 > β2 > α receptor agonist	Yes	Increased [2]
Dopamine	Dose related action on adrenergic and dopaminergic receptors	Yes	Increased [3]
Norepinephrine	Endogenous catecholamine; stimulates β and α adrenergic receptors	Yes	Increased [3]
Milrinone	PDE inhibitor	Yes	Increased [4]
Levosimendan	Calcium sensitizer	No	Not well established [5,6]
Omecamtiv Mecarbil	Enhances myosin and actin cross-bridge formation	No	Unknown [7,8]

Na = sodium; K = potassium; PDE = phosphodiesterase.

## 2. Digoxin

Digoxin is one of the positive inotropic agents which improves hemodynamics and does not have a deteriorating effect on blood pressure or heart rate [9]. Digoxin plays a role in suppressing the neurohormonal activation which is helpful in chronic systolic heart failure patients and can be used for long-term therapy [10]. Digoxin was being extensively used for many years until the Digitalis Investigation Group (DIG) trial showed that digoxin has no mortality benefit in this population, but helps reduce the frequency of hospitalization with exacerbation of heart failure symptoms [11]. There are still controversies regarding the role of digoxin in the treatment of HFrEF, as β blockers were not used in the DIG trial. Monitoring the serum concentration of digoxin in patients is important as a level of ≥1.2 ng/mL is associated with increased mortality. Levels between 0.5 and 0.8 ng/mL are recommended in these patients [12]. *Post hoc* subgroup analyses of the DIG trial showed that women with HFrEF treated with digoxin had an increased mortality [12,13,14,15,16]. However, one of these studies showed that women with a left ventricular ejection fraction (LVEF) less than 35%, and a serum digoxin concentration between 0.5 and 1.1 ng/mL, did not have increased mortality and had reduced hospitalization for heart failure symptoms. Currently, the use of digoxin for treatment of HFrEF in patients symptomatic despite optimal medical therapy has a class IIa indication in the American College of Cardiology/American Heart Association 2013 heart failure guidelines [17], and a class IIb indication for treatment of HFrEF in patients symptomatic despite optimal medical therapy in the European Society of Cardiology heart failure guidelines [18].

Atrial fibrillation is seen commonly in patients with chronic systolic heart failure due to chamber dilation and functional valvular regurgitation. Digoxin plays an important role in obtaining rate control in such patients, as nondihydropyridine calcium channel blockers, such as diltiazem and verapamil, increase mortality in patients with HFrEF [19]. β Blockers are very effective in treating patients with atrial fibrillation associated with HFrEF [20]. However, they cannot be used if these patients are hypotensive or in shock.

Digoxin is a cardiac glycoside with positive inotropic characteristics. It works by inhibiting the sodium-potassium adenosine triphosphatase (ATPase) pump at the cellular level and prevents the transport of sodium from the intracellular to the extracellular space. This process in turn affects the activity of the sodium-calcium pump and raises the intracellular level of calcium by decreasing its efflux, which is responsible for the inotropic effect of the drug. Administration of digoxin has been shown to help in weaning off the mechanical circulatory support and inotropic agents in patients with left ventricular dysfunction [21]. The Randomized Assessment of Digoxin on Inhibitors of Angiotensin-Converting Enzyme (RADIANCE) trial showed that stable chronic heart failure patients with systolic dysfunction had worsening of symptoms when digoxin was withdrawn [1].

## 3. Dopamine

Dopamine is an endogenous catecholamine, and its effects are dose-dependent in patients with cardiogenic shock in the setting of severe left ventricular dysfunction. At low doses (<3 µg/kg/min), dopamine causes vasodilation in the body vasculature including the coronary and renal arteries. The role of low-dose dopamine in helping improve renal function is still not well proven. No improvement was seen in serum creatinine in a cohort of critically ill patients [22]. Neither low-dose dopamine nor low-dose nesiritide improved renal function or symptoms when used in patients hospitalized with acute heart failure and renal dysfunction [23]. An intermediate dose of dopamine (3 to 10 µg/kg/min) not only exerts inotropic and chronotropic effects on the heart but also produces an undesirable elevation in pulmonary capillary wedge pressure (PCWP). At higher infusion rates (10 to 20 µg/kg/min), alpha-receptor mediated vasoconstriction dominates, increasing the afterload, which is unfavorable in the treatment of patients with severe left ventricular dysfunction.

## 4. Dobutamine

Dobutamine is a catecholamine with β-1 and β-2 adrenergic agonist properties which help improve myocardial contractility. In patients with cardiogenic shock due to decompensated heart failure, dobutamine decreases left ventricular end-diastolic pressure and raises blood pressure by increasing cardiac output. In some patients, it can induce hypotension by peripheral vasodilation due to its effect on β-2 receptors. Several studies show improvement in heart failure symptoms with use of dobutamine at continuous infusion doses of 5 to 7.5 µg/kg/min. There is increased mortality associated with dobutamine so it should be used only for in-patient management of patients admitted with decompensated systolic heart failure for improvement in diuresis and symptoms. β blockers including carvedilol and metoprolol are widely used in heart failure patients with left ventricular dysfunction. Carvedilol can mask the inotropic effect of dobutamine more than does metoprolol [24].

Dobutamine is known to cause eosinophilic myocarditis and peripheral eosinophilia. This hypersensitivity reaction is not uncommon in patients awaiting heart transplant, while they are on dobutamine [25]. A small study randomized end-stage heart failure patients to intermittent low-dose dobutamine infused at 2.5 microgram/kilogram/min for 48 h per week for a period of 6 months or optimal medical management. No significant difference was seen in mortality or improvement in symptoms, but all-cause hospitalizations for heart failure exacerbations were lower in the dobutamine group [26]. In the past, dobutamine infusion was used for longer periods of time including as a home inotropic agent. However, in a recent meta-analysis, dobutamine was shown to be associated with higher in-hospital mortality and readmission rates for heart failure exacerbation when compared to nesiritide therapy [2]. Higher doses of dobutamine is not preferred in patients with recent myocardial ischemia, as it can increase myocardial oxygen demand and induce tachycardia. Dobutamine can also be pro-arrhythmic. The half-life of dobutamine is a few minutes, which is an advantage compared to other inotropic agents.

## 5. Norepinephrine 

Norepinephrine is an endogenous catecholamine which is synthesized in the body and carries adrenergic properties. It has both α and β agonist properties. Therefore, it can increase cardiac chronotropic and inotropic response along with peripheral vasoconstriction. It is widely used in patients with septic shock. Due to its β-agonist properties, norepinephrine can lead to tachycardia, which in turn can be harmful in patients with a recent myocardial infarction as it can increase myocardial oxygen demand. The risk of cardiac arrhythmia is also high with the use of norepinephrine. Similar to other vasopressor agents, norepinephrine should be given via a central venous catheter as skin necrosis and sloughing can occur if given via a peripheral intravenous line. 

Norepinephrine use in patients with shock has been compared to dopamine [3]. No mortality benefit was seen in both groups. More side effects such as cardiac arrhythmias were seen with dopamine. A sub-analysis of patients with cardiogenic shock derived more survival benefit from norepinephrine compared to dopamine [3]. Norepinephrine is typically infused at 0.2 to 1 μg/kg/min and is preferred over the use of epinephrine in cardiogenic shock, as epinephrine can promote thrombosis in the coronary vasculature. Patients who present with cardiogenic shock are also occasionally in a vasodilated state causing hypotension. Norepinephrine should be used in such conditions. Patients who have a recent implantation of mechanical circulatory support for end-stage heart failure can also benefit from such a vasoactive agent when they have a component of vasodilatory shock post implant [27].

## 6. Milrinone

Milrinone is a widely used positive inotropic agent in patients with end-stage heart failure and cardiogenic shock [28]. It has been in use for almost two decades. Along with its inotropic properties, milrinone also causes peripheral vasodilation [29]. Milrinone also reduces left ventricular filling pressure in chronic heart failure patients. Milrinone is a bipyridine and inhibits the phosphodiesterase-3 intracellular enzyme, thus preventing the degradation of cyclic adenosine monophosphate (cAMP) within the cell. Increased cAMP levels increase activation of protein kinase A, which in turn leads to more influx of calcium into the cell. Increase in intracellular calcium stimulates myocardial contractility. The positive inotropic action of milrinone is independent of β-receptor stimulation in the myocardial cells, which makes it different from dobutamine and dopamine. This mechanism of action makes use of milrinone preferable in advanced heart failure patients who are on β blockers as part of optimal medical therapy. Milrinone may help reduce pulmonary artery pressure by vasodilating pulmonary vasculature via cAMP, which may improve right ventricular function. Effects of milrinone in pulmonary hypertension and right ventricular failure are still under investigation.

Milrinone is an intravenous preparation that is cleared renally and has a longer half-life of a few hours [30]. It should be avoided in patients with renal failure. Milrinone can induce cardiac arrhythmias and hypotension. These side effects can persist for a few hours even after its infusion is switched off. Initial studies with milrinone showed a reduction in re-hospitalization frequency and improvement in heart failure symptoms [31].

The Outcomes of Prospective Trial of Intravenous Milrinone for Exacerbations of Chronic Heart Failure (OPTIME-CHF) was a randomized controlled trial looking at clinical outcomes when intravenous milrinone was added to standard medical therapy in patients hospitalized with acute exacerbation of heart failure [4]. A total of 951 patients with a mean LVEF of 23% were randomly assigned to either intravenous milrinone or placebo for 48 h and followed for 60 days. Milrinone was associated with significant sustained hypotension and atrial arrhythmias compared to placebo. No differences were seen in in-hospital mortality, 60-day mortality, or readmission [4]. A *post hoc* analysis of patients randomized in the OPTIME-CHF study showed that intravenous milrinone (0.5 µg/kg/min without a loading dose) was associated with higher mortality and re-hospitalization rate in ischemic cardiomyopathy patients [32]. A neutral beneficial effect was seen in patients with non-ischemic cardiomyopathy as the etiology for decompensated heart failure [32]. The Acute Decompensated Heart Failure National Registry (ADHERE) registry also showed a significantly higher in-hospital mortality in patients admitted with acute decompensated heart failure, when treated with milrinone or dobutamine compared to intravenous nitroglycerine or nesiritide [33]. In the Prospective Randomized Milrinone Survival Evaluation (PROMISE) trial, 1088 patients with severe chronic heart failure and left ventricular dysfunction were randomized to oral milrinone or placebo to determine the effect of milrinone on the mortality of such patients who continue to be symptomatic on optimal medical therapy [34]. All patients had symptoms of New York Heart Association functional class III or IV for at least three months. A six-month follow-up showed significantly higher mortality and more frequent hospitalizations in the milrinone group [34]. A meta-analysis of 21 randomized trials also showed that phosphodiesterase inhibitors are associated with significantly higher mortality and cardiac arrhythmias when compared to placebo [35].

Considering the concern for cardiac arrhythmias and higher mortality with milrinone and dobutamine, these inotropic agents should be used in selective groups of patients. They can be considered in cardiogenic shock and decompensated heart failure patients who are not able to be adequately diuresed secondary to worsening end-organ function [36]. Inotropic therapy with milrinone or dobutamine can be used in chronic heart failure patients as a bridge to recovery from an acute hemodynamically compromised state and in advanced stage heart failure patients awaiting advanced heart failure therapies, such as mechanical circulatory support and heart transplant. Milrinone or dobutamine can also be used in stage D heart failure patients who are not candidates for advanced heart failure therapies as a palliative therapy in addition to maximum tolerated optimal medical therapy for symptom improvement.

## 7. Levosimendan

Levosimendan is a calcium sensitizing agent which can exert its inotropic effect by increasing the sensitivity of cardiomyocyte to intracellular calcium. Levosimendan increases the sensitivity of cardiomyocyte to intracellular calcium by binding to troponin C. Achieving an inotropic effect without increasing intracellular calcium levels can prevent an increased risk of cardiac arrhythmia with this agent. Levosimendan also has vasodilatory properties by opening adenosine triphosphate (ATP)-sensitive potassium channels in vascular smooth muscle, causing their relaxation. This mechanism reduces the preload and afterload which is helpful in treating patients with acute decompensated heart failure. It may also have some phosphodiesterase (PDE) inhibitor activity. Levosimendan is widely used in Europe but is not approved for this use in the United States.

Short-term use of levosimendan has been shown to cause rapid dose-dependent improvement in hemodynamics and symptoms in patients with decompensated heart failure [37,38]. In the Levosimendan Infusion *versus* Dobutamine (LIDO) study, intravenous levosimendan was compared with dobutamine in severe low-output heart failure patients [39]. A hemodynamic improvement (increase in cardiac output and decrease in PCWP) was associated with a lower mortality at one- and six-months with levosimendan compared to dobutamine [39]. Similarly, in the Randomized Study on Safety and Effectiveness of Levosimendan in Patients with Left Ventricular Failure after an Acute Myocardial Infarct (RUSSLAN) trial, levosimendan did not cause hypotension or clinically significant ischemia [40]. Levosimendan also reduced the risk of worsening heart failure and death [40]. In the Survival of Patients with Acute Heart Failure in Need of Intravenous Inotropic Support (SURVIVE) trial, levosimendan failed to significantly reduce all-cause mortality at 180 days and did not affect any secondary clinical outcomes, when compared to dobutamine [5]. The Randomized Multicenter Evaluation of Intravenous Levosimendan Efficacy (REVIVE-II) study showed that levosimendan was associated with more adverse effects like hypotension and cardiac arrhythmias while providing improvement in symptoms in acutely decompensated heart failure patients [6]. Recent European Society of Cardiology (ESC) guidelines for the treatment of patients with acute heart failure recommend that intravenous levosimendan may be considered to reverse the state of hypoperfusion caused by β-blockers [18]. Although these trials are available, the safety and clinical efficacy of levosimendan has not been well established in these patients.

## 8. Omecamtiv Mecarbil

Due to multiple risks associated with current inotropes being used in clinical practice, there have been many efforts in the last few years to develop safe and efficacious inotropes. Current inotropic agents increase cAMP which results in increased levels of intracellular calcium (Table 1). A high level of calcium in cardiac myocytes is associated with many risks, and its homeostasis requires more energy utilization via adenosine triphosphate (ATP). This increases myocardial oxygen demand which is not beneficial in patients being treated for heart failure. Omecamtiv mecarbil is one of the newer agents and works as a cardiac specific myosin activator [41]. Omecamtiv mecarbil exerts an inotropic property resulting in the improvement of systolic function of the heart without increasing its energy demand. This drug has been studied in patients with heart failure [41].

Cardiac myocytes contract through an interaction between the myofilaments actin and myosin. The chemical energy derived from this cross-bridge formation between myofilaments is derived from the breakdown of ATP. Omecamtiv mecarbil activates the myocardial ATPase, thereby causing an effective interaction between myofilaments possible. This increases the contractile force, hence improving the stroke volume. A phase II trial investigated the use of omecamtiv mecarbil in stable heart failure patients with left ventricular dysfunction. Increase in left ventricular ejection fraction and stroke volume was seen with reduction in left ventricular end-systolic and end-diastolic volumes [7]. In another crossover study, omecamtiv mecarbil caused improvement in stroke volume and left ventricular ejection [8].

**Figure 1:**
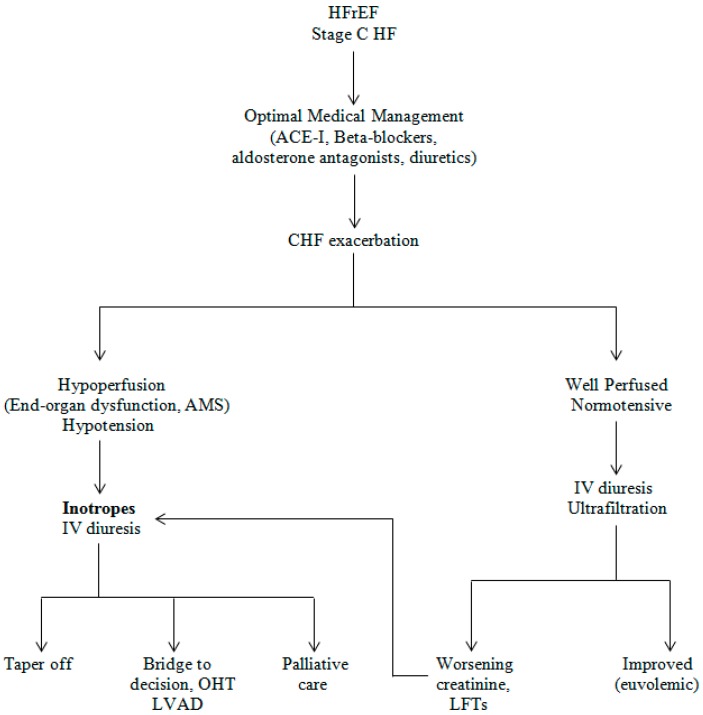
Approach to use of inotropic agents in patients hospitalized with acute decompensated systolic heart failure. HFrEF = Heart failure with reduced ejection fraction; ACE-I = Angiotensin converting enzyme inhibitor; CHF = congestive heart failure; AMS = Altered mental status; IV = intravenous; OHT = Orthotropic heart transplant; LVAD = Left ventricular assist device; LFT = Liver function tests.

## 9. Selection of Patients for Home Inotropes

Long-term inotropic therapy (milrinone or dobutamine) should be offered in selected patients. A detailed conversation with the patient and family shall be held, including a discussion on the risks and benefits of use of inotropes. Inotropes at home can be managed with the help of a home infusion program and a visiting nurse. Chronic heart failure patients awaiting heart transplants are candidates for intravenous inotropic support until the donor heart becomes available [42]. This helps to maintain hemodynamic stability and keep the fluid status and pulmonary pressures optimized prior to the surgery. On the other hand, in patients with severe heart failure who are not candidates for advanced heart failure therapies, such as transplant and mechanical circulatory support, inotropic agents can be used for palliative therapy (Figure 1) [43]. In such a situation, the patient and family should be informed of the risks and benefits of this therapy. Inotropes can help reduce frequency of hospitalizations and improve symptoms in these patients.

## References

[1] Packer M., Gheorghiade M., Young J.B., Costantini P.J., Adams K.F., Cody R.J., Smith L.K., van Voorhees L., Gourley L.A., Jolly M.K. (1993). Withdrawal of digoxin from patients with chronic heart failure treated with angiotensin-converting-enzyme inhibitors: RADIANCE Study. N. Engl. J. Med..

[2] Wang X.C., Zhu D.M., Shan Y.X. (2015). Dobutamine therapy is associated with worse clinical outcomes compared with nesiritide therapy for acute decompensated heart failure: A systematic review and meta-analysis. Am. J. Cardiovasc. Drugs.

[3] De Backer D., Biston P., Devriendt J., Madl C., Chochrad D., Aldecoa C., Brasseur A., Defrance P., Gottignies P., Vincent J.L. (2010). Comparison of dopamine and norepinephrine in the treatment of shock. N. Engl. J. Med..

[4] Cuffe M.S., Califf R.M., Adams K.F., Benza R., Bourge R., Colucci W.S., Massie B.M., O’Connor C.M., Pina I., Quigg R. (2002). Short-term intravenous milrinone for acute exacerbation of chronic heart failure: A randomized controlled trial. JAMA.

[5] Mebazaa A., Nieminen M.S., Packer M., Cohen-Solal A., Kleber F.X., Pocock S.J., Thakkar R., Padley R.J., Põder P., Kivikko M. (2007). Levosimendan vs. dobutamine for patients with acute decompensated heart failure: The SURVIVE Randomized Trial. JAMA.

[6] Packer M., Colucci W., Fisher L., Massie B.M., Teerlink J.R., Young J., Padley R.J., Thakkar R., Delgado-Herrera L., Salon J. (2013). Effect of levosimendan on the short-term clinical course of patients with acutely decompensated heart failure. J. Am. Coll. Cardiol. Heart Fail..

[7] Cleland J.G., Teerlink J.R., Senior R., Nifontov E.M., Mc Murray J.J., Lang C.C., Tsyrlin V.A., Greenberg B.H., Mayet J., Francis D.P. (2011). The effects of the cardiac myosin activator, omecamtiv mecarbil, on cardiac function in systolic heart failure: A double-blind, placebo-controlled, crossover, dose-ranging phase 2 trial. Lancet.

[8] Teerlink J.R., Clarke C.P., Saikali K.G., Lee J.H., Chen M.M., Escandon R.D., Elliott L., Bee R., Habibzadeh M.R., Goldman J.H. (2011). Dose-dependent augmentation of cardiac systolic function with the selective cardiac myosin activator, omecamtiv mecarbil: A first-in-man study. Lancet.

[9] Gheorghiade M., St Clair J., St Clair C., Beller G.A. (1987). Hemodynamic effects of intravenous digoxin in patients with severe heart failure initially treated with diuretics and vasodilators. J. Am. Coll. Cardiol..

[10] Gheorghiade M., Braunwald E. (2009). Reconsidering the role for digoxin in the management of acute heart failure syndromes. JAMA.

[11] The Digitalis Investigation Group (1997). The effect of digoxin on mortality and morbidity in patients with heart failure. N. Engl. J. Med..

[12] Rathore S.S., Curtis J.P., Wang Y., Bristow M.R., Krumholz H.M. (2003). Association of serum digoxin concentration and outcomes in patients with heart failure. JAMA.

[13] Rathore S.S., Wang Y., Krumholz H.M. (2002). Sex-based differences in the effect of digoxin for the treatment of heart failure. N. Engl. J. Med..

[14] Adams K.F., Patterson J.H., Gattis W.A., O’Connor C.M., Lee C.R., Schwartz T.A., Gheorghiade M. (2005). Relationship of serum digoxin concentration to mortality and morbidity in women in the digitalis investigation group trial: A retrospective analysis. J. Am. Coll. Cardiol..

[15] Ahmed A., Aronow W.S., Fleg J.L. (2006). Predictors of mortality and hospitalization in women with heart failure in the Digitalis Investigation Group trial. Am. J. Ther..

[16] Ahmed A., Aban I.B., Weaver M.T., Aronow W.S., Fleg J.L. (2006). Serum digoxin concentration and outcomes in women with heart failure: A bi-directional effect and a possible effect modification by ejection fraction. Eur. J. Heart Fail..

[17] Yancy C.W., Jessup M., Bozkurt B., Butler J., Casey D.E., Drazner M.H., Fonarow G.C., Geraci S.A., Horwich T., Januzzi J.L. (2013). 2013 ACCF/AHA guideline for the management of heart failure: A report of the American College of Cardiology Foundation/American Heart Association Task Force on Practice Guidelines. J. Am. Coll. Cardiol..

[18] McMurray J.J., Adamopoulos S., Anker S.D., Auricchio A., Böhm M., Dickstein K., Falk V., Filippatos G., Fonseca C., Gomez-Sanchez M.A. (2012). ESC guidelines for the diagnosis and treatment of acute and chronic heart failure 2012: The Task Force for the Diagnosis and Treatment of Acute and Chronic Heart Failure 2012 of the European Society of Cardiology. Developed in collaboration with the Heart Failure Association (HFA) of the ESC. Eur. J. Heart Fail..

[19] Goldstein R.E., Boccuzzi S.J., Cruess D., Nattel S. (1991). Diltiazem increases late-onset congestive heart failure in postinfarction patients with early reduction in ejection fraction. Circulation.

[20] Aronow W.S. (2009). Management of atrial fibrillation in the elderly. Minerva Med..

[21] Naqvi S., Ahmed I., Siddiqi R., Hussain S.A. (2010). Digoxin as a rescue drug in intra aortic balloon pump and inotrope dependent patients. J. Ayub. Med. Coll. Abbottabad.

[22] Bellomo R., Chapman M., Finfer S., Hickling K., Myburgh J. (2000). Low dose dopamine in patients with early renal dysfunction: A placebo controlled randomised trial. Australian and New Zealand Intensive Care Society (ANZICS) Clinical Trials Group. Lancet.

[23] Chen H.H., Anstrom K.J., Givertz M.M., Stevenson L.W., Semigran M.J., Goldsmith S.R., Bart B.A., Bull D.A., Stehlik J., LeWinter M.M. (2013). Low-dose dopamine or low-dose nesiritide in acute heart failure with renal dysfunction: The ROSE acute heart failure randomized trial. JAMA.

[24] Metra M., Nodari S., D’Aloia A., Muneretto C., Robertson A.D., Bristow M.R., Dei Cas L. (2002). β-Blocker therapy influences the hemodynamic response to inotropic agents in patients with heart failure: A randomized comparison of dobutamine and enoximone before and after chronic treatment with metoprolol or carvedilol. J. Am. Coll. Cardiol..

[25] Takkenberg J.J., Czer L.S., Fishbein M.C., Luthringer D.J., Quartel A.W., Mirocha J. (2004). Eosinophilic myocarditis in patients awaiting heart transplantation. Crit. Care Med..

[26] Oliva F., Latini R., Politi A., Staszewsky L., Maggioni A.P., Nicolis E., Mauri F. (1999). Intermittent 6-month low-dose dobutamine infusion in severe heart failure: DICE multicenter trial. Am. Heart J..

[27] Jolly S., Newton G., Horlick E., Seidelin P.H., Ross H.J., Husain M., Dzavik V. (2005). Effect of vasopressin on hemodynamics in patients with refractory cardiogenic shock complicating acute myocardial infarction. Am. J. Cardiol..

[28] Overgaard C.B., Dzavik V. (2008). Inotropes and vasopressors: Review of physiology and clinical use in cardiovascular disease. Circulation.

[29] Colucci W.S., Wright R.F., Jaski B.E., Fifer M.A., Braunwald E. (1986). Milrinone and dobutamine in severe heart failure: Differing hemodynamic effects and individual patient responsiveness. Circulation.

[30] Cox Z.L., Calcutt M.W., Morrison T.B., Akers W.S., Davis M.B., Lenihan D.J. (2013). Elevation of plasma milrinone concentrations in stage D heart failure associated with renal dysfunction. J. Cardiovasc. Pharmacol. Ther..

[31] Marius-Nunez A.L., Heaney L., Fernandez R.N., Clark W.A., Ranganini A., Silber E., Denes P. (1996). Intermittent inotropic therapy in an outpatient setting: A cost-effective therapeutic modality in patients with refractory heart failure. Am. Heart J..

[32] Felker G.M., Benza R.L., Chandler A.B., Leimberger J.D., Cuffe M.S., Califf R.M., Gheorghiade M., O’Connor C.M., OPTIME-CHF Investigators (2003). Heart failure etiology and response to milrinone in decompensated heart failure: Results from the OPTIME-CHF study. J. Am. Coll. Cardiol..

[33] Abraham W.T., Adams K.F., Fonarow G.C., Costanzo M.R., Berkowitz R.L., LeJemtel T.H., Cheng M.L., Wynne J., ADHERE Scientific Advisory Committee and Investigators, ADHERE Study Group (2005). In-hospital mortality in patients with acute decompensated heart failure requiring intravenous vasoactive medications: An analysis from the Acute Decompensated Heart Failure National Registry (ADHERE). J. Am. Coll. Cardiol..

[34] Packer M., Carver J.R., Rodeheffer R.J., Ivanhoe R.J., DiBianco R., Zeldis S.M., Hendrix G.H., Bommer W.J., Elkayam U., Kukin M.L. (1991). Effect of oral milrinone on mortality in severe chronic heart failure. The PROMISE Study Research Group. N. Engl. J. Med..

[35] Amsallem E., Kasparian C., Haddour G., Boissel J.P., Nony P. (2005). Phosphodiesterase III inhibitors for heart failure. Cochrane Database Syst. Rev..

[36] Dec G.W. (2005). Acute decompensated heart failure: The shrinking role of inotropic therapy. J. Am. Coll. Cardiol..

[37] Nieminen M.S., Akkila J., Hasenfuss G., Kleber F.X., Lehtonen L.A., Mitrovic V., Nyquist O., Remme W.J. (2000). Hemodynamic and neurohumoral effects of continuous infusion of levosimendan in patients with congestive heart failure. J. Am. Coll. Cardiol..

[38] Slawsky M.T., Colucci W.S., Gottlieb S.S., Greenberg B.H., Haeusslein E., Hare J., Hutchins S., Leier C.V., LeJemtel T.H., Loh E. (2000). Acute hemodynamic and clinical effects of levosimendan in patients with severe heart failure. Study Investigators. Circulation.

[39] Follath F., Cleland J.G., Just H., Papp J.G., Scholz H., Peuhkurinen K., Harjola V.P., Mitrovic V., Abdalla M., Sandell E.P. (2002). Efficacy and safety of intravenous levosimendan compared with dobutamine in severe low-output heart failure (the LIDO study): A randomised double-blind trial. Lancet.

[40] Moiseyev V.S., Põder P., Andrejevs N., Ruda M.Y., Golikov A.P., Lazebnik L.B., Kobalava Z.D., Lehtonen L.A., Laine T., Nieminen M.S. (2002). Safety and efficacy of a novel calcium sensitizer, levosimendan, in patients with left ventricular failure due to an acute myocardial infarction. A randomized, placebo-controlled, double-blind study (RUSSLAN). Eur. Heart J..

[41] Teerlink J.R. (2009). A novel approach to improve cardiac performance: Cardiac myosin activators. Heart Fail. Rev..

[42] Brozena S.C., Twomey C., Goldberg L.R., Desai S.S., Drachman B., Kao A., Popjes E., Zimmer R., Jessup M. (2004). A prospective study of continuous intravenous milrinone therapy for status IB patients awaiting heart transplant at home. J. Heart Lung Transplant..

[43] López-candales A.L., Carron C., Schwartz J. (2004). Need for hospice and palliative care services in patients with end-stage heart failure treated with intermittent infusion of inotropes. Clin. Cardiol..

